# Adsorption of Phenol on Commercial Activated Carbons: Modelling and Interpretation

**DOI:** 10.3390/ijerph17030789

**Published:** 2020-01-28

**Authors:** Bingxin Xie, Jihong Qin, Shu Wang, Xin Li, Hui Sun, Wenqing Chen

**Affiliations:** 1 Department of Environmental Science and Engineering, Sichuan University, Chengdu 610065, China; bingxinx@foxmail.com (B.X.); w.dashu@foxmail.com (S.W.); lixinscu@outlook.com (X.L.); cwq69814@126.com (W.C.); 2 Department of Environmental Engineering, Chengdu University, Chengdu 610106, China; qinjihongcdu@foxmail.com

**Keywords:** activated carbons, adsorbent, phenol, adsorption

## Abstract

Adsorption by activated carbons (AC) is an effective option for phenolic wastewater treatment. Three commercial AC, including coal-derived granular activated carbons (GAC_950_), coal-derived powdered activated carbons (PAC_800_), and coconut shell-derived powdered activated carbons (PAC_1000_), were utilized as adsorbent to study its viability and efficiency for phenol removal from wastewater. Pseudo-first order, pseudo-second order, and the Weber–Morris kinetic models were used to find out the kinetic parameters and mechanism of adsorption process. Further, to describe the equilibrium isotherms, the experimental data were analyzed by the Langmuir and Freundlich isotherm models. According to the experimental results, AC presented a micro/mesoporous structure, and the removal of phenol by AC was affected by initial phenol concentration, contact time, pH, temperature, and humic acid (HA) concentration. The pseudo-second order kinetic and Langmuir models were found to fit the experimental data very well, and the maximum adsorption capacity was 169.91, 176.58, and 212.96 mg/g for GAC_950_, PAC_800_, and PAC_1000_, respectively, which was attributed to differences in their precursors and physical appearance. Finally, it was hard for phenol to be desorbed in a natural environment, which confirmed that commercial AC are effective adsorbents for phenol removal from effluent wastewater.

## 1. Introduction

Activated carbons (AC) are carbonaceous materials with large specific surface area, superior porosity, high physicochemical-stability, and excellent surface reactivity, extensively used for adsorption of several environmental contaminants, gas separation, heterogeneous catalysis, gas storage, and gas masks, among others [1]. The worldwide demand of AC reached 12,804,000 tons in 2015, and the research into AC has also had an important increase, yielding a production of 17,516 research papers from 1995 to 2016 [2]. Almost all carbon-rich precursors can be converted to AC through stabilization (if required), carbonization, and activation [3]. Selection of the raw material depends not only on the anticipated role of carbon-surface functionalities for given applications, but on the availability and low cost of raw material. In China, coal and coconut shell are the most common precursors for the largescale synthesis of commercial AC. Moreover, there are two most common physical forms, in which AC are used, these being granular active carbons (GAC) and powdered active carbons (PAC) [4]. Various commercial AC with different properties are manufactured for different applications.

Phenol is an organic compound found in wastewater disposed from many industries, such as refinery, petrochemical, coal processing, pharmaceutical, polymeric resin, and pesticide industries, among others [5,6]. Currently, phenol is produced at a rate of about 6 million ton/year worldwide, with a significantly increasing trend [7]. Excessive inhalation or exposure to phenol can cause coma, convulsions, cyanosis, and other adverse reactions [5,7]. Phenol has also been registered as a priority pollutant by the US Environmental Protection Agency (USEPA), with a permissible limit of 0.1 mg/L in wastewater and 1 ug/mL in water supplies [8]. Several technologies, including adsorption [9], oxidation [10], membrane separation [11], biodegradation [12], and ion exchange [13], have been proposed for treatment of the phenol wastewaters. Among them, adsorption by AC is the most widely used and most powerful technique for the elimination of phenol.

The adsorption capacity and mechanisms of phenol adsorption by AC could be influenced by the properties of AC, such as specific surface area, surface functional groups, pore size distribution, and other surface characteristics. For example, Lorenc-Grabowska et al. (2016) [14] reported that the main mechanism determining phenol adsorption was micropore filling within a pore size of 0.8-1.4 nm. Zhang et al. (2016) [9] found that phenol adsorption capacity improved in comparison with the raw sample, owing to the decrease of total oxygen-containing functional groups on the thermal modified AC samples. The properties of AC are not only strongly dependent on AC feedstocks, but also on physical appearance. Despite vast studies on factors that influence the process of phenol uptake, there are relatively few studies focusing on the comparison of adsorption mechanisms for commercial AC produced from different feedstocks and different physical appearance. Another important aspect is that once the activated carbon is exhausted, it must be carefully disposed as hazardous waste. Various technologies including thermal regeneration [15], chemical regeneration [16], bio-regeneration [4], and ultrasound [17] can be used for the desorption of phenols from AC. However, the release mechanism of phenol under natural conditions has not been fully understood up until now. Therefore, the objectives of our work are (1) to investigate impacts of feedstock and physical appearance on the properties of commercial AC, as well as their difference of adsorption capacity of phenol; (2) to optimize phenol adsorption condition, kinetic model, and isotherm model for commercial AC; and (3) to determine desorption characteristics of the three commercial AC under different release conditions.

## 2. Materials and Methods

### 2.1. Materials

The commercial activated carbons were collected from Sichuan Nan-Ke Activated Carbon Co., Ltd (Chengdu, China) and were classified as GAC_950_, PAC_800_, and PAC_1000_, where GAC and PAC represent granular active carbons and powdered activated carbons, respectively, and the last characters 950, 800, and 1,000 represent the iodine values of activated carbons. In addition, GAC_950_ and PAC_800_ were produced by coal, whereas PAC_1000_ was produced by coconut shell. All of the AC were washed thoroughly with deionized water and ethanol to remove any water-soluble impurities and then dried at 110 °C. Phenol, 4-aminoantipyrine, potassium ferricyanide, and other relevant solvents were purchased from Chengdu Ke-Long Chemical Regent Co. (Chengdu, China). All chemical reagents were analytical grade and used directly without further purification.

### 2.2. Physical and Chemical Characterization of AC

The surface morphologies of AC were observed by scanning electron microscope (SEM, Hitachi SU3500, Tokyo, Japan) after coating with gold for 30 s. The AC textural properties (specific surface area (As), average pore size, and total pore volume) were determined on the basis of nitrogen adsorption/desorption isotherms at 77 K, using a Micrometrics Gemini 2390 system. The specific surface area was determined with the standard Brunauer–Emmett–Teller (BET) method and cumulative pore volumes by Barrett–Joyner–Halenda analysis. Fourier transform infrared (FTIR) spectroscopy (Tracer-100, Tokyo, Japan) was applied to determine the surface chemistry of the AC. All the samples and the dried KBr were ground at a mass ratio of 1:100, and the spectra were recorded from 400 to 4000 cm^−1^. The pH_PZC_ (point of zero charge) of AC was determined by mixing 1 g of each AC with 20 mL of CO_2_-free deionized water, according to the procedure described by Moreno-Castilla et al. (2000) [18].

### 2.3. Phenol Adsorption on AC

Several key parameters, including the initial concentration (10–400 mg/L), contact time (0–400 min), pH (3–11), temperature (20–60 °C), and the concentration of humid acid (HA, 0–100 mg/L) were systematically taken into account to optimize the adsorption conditions. A total of 50 mg of AC and 80 mL of phenol solution were added to polyethylene tubes, which were placed onto a water bath oscillator at 150 r/min. Afterwards, the carbons were filtered and the concentration of phenol was analyzed by 4-aminoantipyrine spectrophotometric method. Phenol solution (1 mL) was taken in a clean dry colorimetric tube, then the volume was fixed to 50 mL with deionized water. The pH of solution was adjusted by adding 0.5 mL buffer solution (20% ammonia-ammonium chloride solution), and then 1 mL of 4-aminoantipyrine and 1 mL of potassium ferricyanide were added and mixed well. In this method, phenol reacts with 4-aminoantipyrine in the presence of potassium ferricyanide to form a colored antipyrine dye [19,20]. The colored sample was measured by UV-1100 UV-visible spectrophotometer (Shanghai Mapada Instrument Co. Ltd., Shanghai, China) at 510 nm. The assays were performed in replicate (*n* = 3) and blank tests were realized. The adsorption percentage (R, %) and the amount of phenols (q_e_, mg/g) on AC at equilibrium were calculated according to Equations (1) and (2):

(1) $$q_{e}=\frac{V(C_{0}-C_{e})}{m}$$

(2) $$R(\%)=\frac{\left(C_{0}-C_{e}\right)}{C_{0}}\times 100\%$$

where C_0_ (mg/L) and C_e_ (mg/L) are the initial and equilibrium concentrations of phenol, respectively; V (L) is the volume of the phenol solution; and m (g) is the amount of AC applied for adsorption.

#### 2.3.1. Adsorption Kinetic Models

The kinetics of adsorption is an important characteristic to define the efficiency of adsorption. In order to investigate the mechanism of adsorption and kinetic parameters, sorption data was analyzed using pseudo-first order (Equation (3)), pseudo-second order (Equation (4)), and the Weber–Morris model (Equation (5)) [21]. Compared with the pseudo-first order and pseudo-second order kinetic models, the Weber–Morris model can generally identify the diffusion mechanism and rate controlling steps affecting the kinetics of adsorption. If an adsorption process is solely governed by intraparticle diffusion, the initial part of the Weber–Morris plot is a straight line passing through the origin. Otherwise, the intraparticle diffusion is not the only rate-controlling step but some degree of the boundary layer diffusion (or external mass transfer) also controls the adsorption [22].

(3) $$\ln(q_{e,\exp}-q_{t})=\ln q_{e}-k_{1}t$$

(4) $$\frac{t}{q_{t}}=\frac{1}{q_{e}^{2}k_{2}}+\frac{t}{q_{e}}$$

(5) $$q_{t}=k_{3}t^{1/2}+C$$

where t (min) and *q_t_* (mg/g) are, respectively, the adsorption time and the amount of phenol adsorbed on AC at any time, t; *q_e,exp_* (mg/g) and q_e_ (mg/g) are the amount of phenol adsorbed at experimental-equilibrium and calculated-equilibrium, respectively; k_1_ (min^−1^), k_2_ (g/mg/min), and k_3_ (mg/g/min^1/2^) are the rate constants of the pseudo-first order, pseudo-second order, and the Weber–Morris models, respectively.

#### 2.3.2. Adsorption Equilibrium Models

The equilibrium adsorption data were fitted by Langmuir (Equation (6)) and Freundlich (Equation (7)) isotherm equations. The Langmuir isotherm is valid for monolayer adsorption onto a surface with a finite number of identical sites, and it is based on the assumption of adsorption homogeneity, such as equally available adsorption sites, monolayer surface coverage, and no interactions between adsorbed species. The Freundlich equation is an empirical relationship in which it is assumed that the non-ideal adsorption takes place on a heterogeneous surface with different adsorption energy and characters. It has been successfully applied for applications involving multilayer adsorption [23,24].

(6) $$\frac{C_{e}}{q_{e}}=\frac{1}{K_{L}q_{m}}+\frac{C_{e}}{q_{m}}$$

(7) $$\log q_{e}=\log K_{F}+\frac{1}{n}\log C_{e}$$

where q_e_ (mg/g) is the amount of phenol adsorbed at equilibrium, C_e_ (mg/L) is the equilibrium concentration of phenol, q_m_ (mg/g) is the maximum adsorption capacity, and K_L_ (L/mg) is the Langmuir adsorption constant. K_F_ (mg/g) and 1/n (unitless) are the Freundlich model constants related to adsorption capacity and adsorption intensity, respectively.

### 2.4. Phenol Desorption on AC

Desorption studies were conducted using various solution (deionized water/acid/alkali) and the equilibration time was 24 h. Nine samples of 50 mg each of AC saturated with phenol at initial concentration of 300 mg/L were prepared. Six AC samples were taken to be used at desorption experiment by NaOH (pH 12) and H_2_SO_4_ (pH 3) solution, whereas the other samples were taken in polyethylene tubes, including only deionized water to determine amount of desorbed phenol by shaker. After equilibrium, the phenol concentration in the solution was measured. The assays were performed in replicate (*n* = 3) and the release percentage (R_d_) was calculated through the following equation:

(8) $$R_{d}(\%)=\frac{C_{d}\times V_{d}}{m\times q_{e}}\times 100\%$$

where C_d_ (mg/L) is the concentration of phenol in release solution; V_d_ (L) is the volume of the release solution, and *V_d_* = 0.08 in this work; m (g) is the amount of AC applied for release; and q_e_ (mg/g) is the amount of phenol on AC at equilibrium.

## 3. Results and Discussion

### 3.1. Characterization of AC

**Figure 1:**
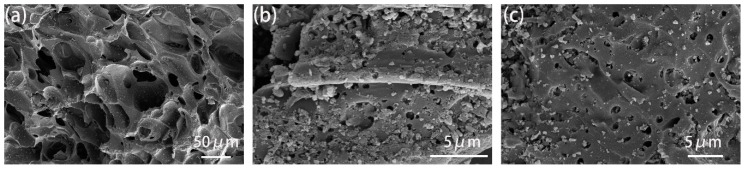
Scanning electron microscope (SEM) images of (**a**) coal-derived granular activated carbons (GAC_950_), (**b**) coal-derived powdered activated carbons (PAC_800_), and (**c**) coconut shell-derived powdered activated carbons (PAC_1000_).

Activated carbon is generally described as an amorphous form of graphite with a random structure of graphite plates, having highly porous structure with a range of cracks and crevices reaching molecular dimensions. As shown in Figure 1, GAC_950_ demonstrated a skeletal structure with pores, whereas PAC_800_ and PAC_1000_ had a uniform morphology. Further, the number of pores in PAC_1000_ were found to be more than that of PAC_800_, and these were also relatively larger in size. The presence of cavities in the AC structure is favorable for the adsorption process because they enable the penetration of phenol molecules into the adsorbent [25,26].

**Figure 2:**
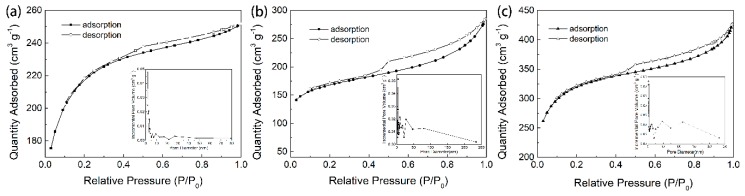
Nitrogen adsorption-desorption isotherms and the Barrette–Joynere–Halenda desorption pore size distribution of (**a**) GAC_950_, (**b**) PAC_800_, and (**c**) PAC_1000_.

Physisorption with inert gas (N_2_) is one of the most common and effective ways to determine the specific surface area of porous materials. All the adsorption isotherms showed a mixture of type I and type IV isotherms. Thereafter, a hysteresis in the desorption of the N_2_ isotherm appeared. This result was consistent with the pore size distribution curve and demonstrated the microporosity to mesoporosity (1.5-50 nm) of AC (Figure 2) [27]. According to the nitrogen isotherms, GAC_950_, PAC_800_, and PAC_1000_ showed high surface areas of 700.57 m^2^/g, 542.33 m^2^/g, and 1025.02 m^2^/g, respectively. The average pore size defines the ability of the adsorbate molecules to penetrate inside the AC; only when the pores have a diameter larger than the effective molecular diameter of the adsorbate, the adsorbate molecules penetrate the adsorbent [28]. Phenol has an effective molecule diameter of 0.75 nm, and the average pore sizes presented by GAC_950_, PAC_800_, and PAC_1000_, were 2.22, 3.25, and 5.01 nm, respectively; all AC were suitable for phenol adsorption.

**Figure 3:**
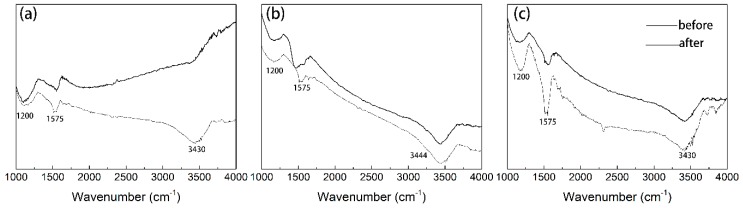
FTIR spectra of (**a**) GAC_950_, (**b**) PAC_800_, and (**c**) PAC_1000_ before and after phenol adsorption.

The functional groups on the AC (before adsorption and after adsorption) were measured by the FTIR spectroscopy, as depicted in Figure 3. The band at 3430 cm^-1^ referred to the O-H functional groups for all AC, and strong absorptions at around 1200 and 1500 cm^-1^ were observed, which might be assigned to C-H stretching of -CH_3_, -CH_2_ groups, and C=O stretching of quinone groups [8,25]. The presence of these oxygenated groups confers a negative charge density in AC surface. The phenol adsorption onto activated carbon occurred through the formation of a donor-acceptor complex between the electron donor groups (e.g., carbonyls) at the AC surface and the aromatic ring of the phenol that acts as the acceptor [14,29]. In addition, the hydroxyl adsorption band widened and a diffuse broad spectrum appeared after adsorption; which suggested that hydrogen bond also favor the phenol adsorption [30].

### 3.2. Kinetics of Phenol Adsorption on AC

**Figure 4:**
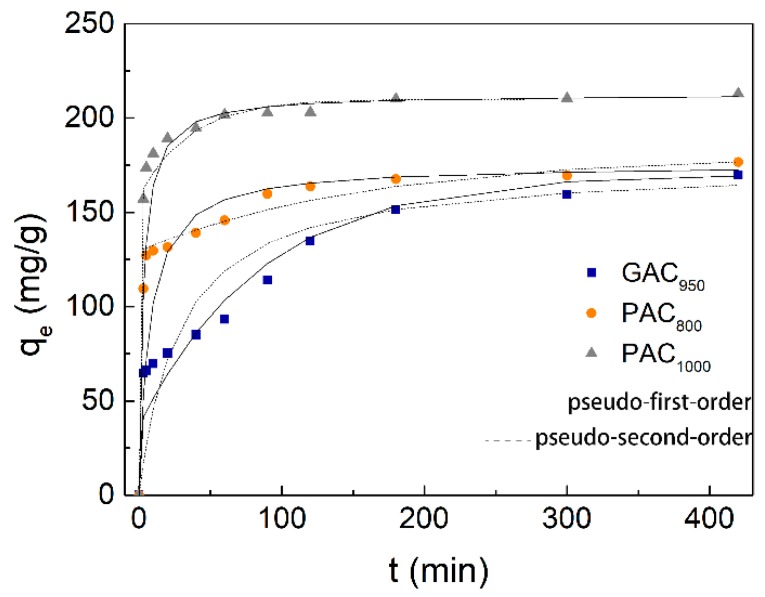
Kinetic curves for phenol adsorption onto AC.

According to previous reports, q_e_ of any adsorbent is highly dependent on the contact time with the adsorbate. The determination of equilibrium time of phenol adsorption was conducted at the initial phenol concentration range of 300 mg/L under neutral pH and temperature of 25 °C, and the experimental result is given in Figure 4. The rate of phenol adsorption was found to be very rapid during the initial 100 min. Thereafter, the adsorption capacity reached a plateau as the contact time increased further and exhibited adsorption at equilibrium after 180 min. This behavior can be explained because a large number of vacant surface sites are available for adsorption during the initial stage, and after a lapse of time, the remaining vacant surface sites are difficult to occupy due to repulsive forces between the solute molecules on the solid and bulk phases [31]. At equilibrium, the q_e,exp_ of GAC_950_, PAC_800_, and PAC_1000_ were 169.91, 176.58, and 212.96 mg/g, respectively. It was also found that the removal of phenol by GAC_950_ was only slightly less than that by PAC_800_ at any contact time and that the removal of phenol by PAC_800_ was around 17% less than that by PAC_1000_.

**Figure 5:**
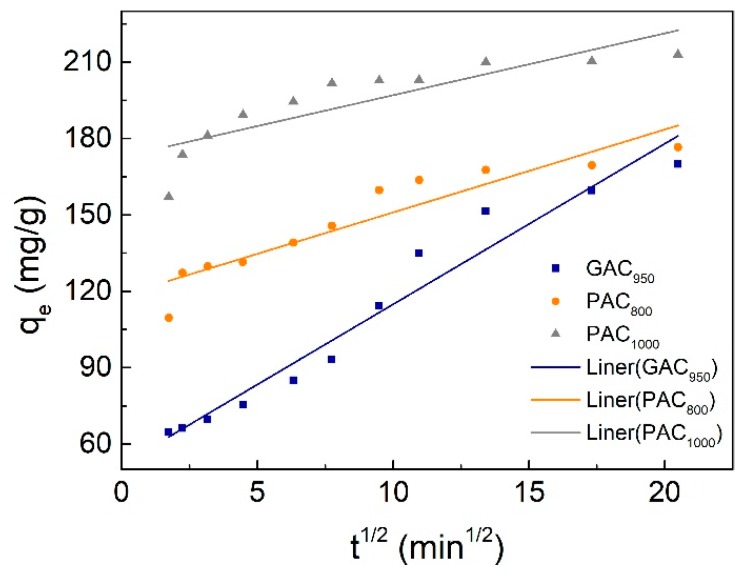
Weber-Morris plot for phenol adsorption onto activated carbons (AC).

Pseudo-first order, pseudo-second order, and the Weber–Morris model kinetic parameters are represented in Table 1. The correlation coefficient for the pseudo-second order was higher than that of pseudo-first order kinetic model for both AC, and the calculated values of q_e_ were extremely close to the experimental data (q_e,exp_), whereas the gap between calculated values and experimental data for pseudo-first-order model was more obvious. This indicated that the adsorption perfectly complied with pseudo-second order reaction and the adsorption of phenol appeared to be controlled by the chemisorption process simultaneously. In other words, the adsorption of phenol happened via surface exchange reactions until the surface functional sites were fully occupied; thereafter, phenol molecules diffused into the AC network for further interactions (such as inclusion complex, hydrogen bonding, hydrogen-phobic interactions) [32]. This result was consistent with the kinetics behavior of phenol adsorption on other AC samples [9,25]. On the other hand, the Weber–Morris plots were shown in Figure 5. The plots were not linear over the whole time range, implying that more than one process affected the adsorption, and the adsorption process contained both the surface adsorption and intraparticle diffusion. These behaviors can be explained due to the presence of micropores in the adsorbent.

### 3.3. Isotherm of Phenol Adsorption on AC

**Figure 6:**
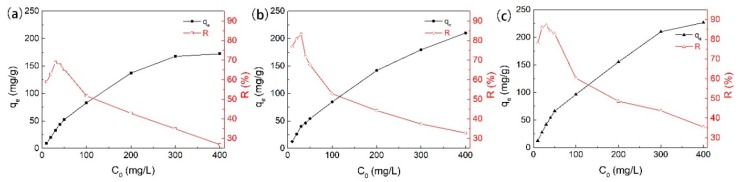
Effects of the initial phenol concentrations on the adsorption on (**a**) GAC_950_, (**b**) PAC_800_, and (**c**) PAC_1000_.

We investigated the impact of initial phenol concentration (C_0_) by fixing adsorbent dose (50 mg) and contact time (≥420 min) under neutral pH and at ambient temperature (25 °C). As shown by the results in Figure 6, the amount of phenol adsorbed per unit weight of adsorbent increased with increasing C_0_. The C_0_ provided the necessary driving force to overcome the resistances to the mass transfer of phenol between aqueous and solid phases, and the increase in C_0_ also enhanced the interaction between phenol and AC [31]. Therefore, an increase in C_0_ of phenol enhanced the adsorption uptake of phenol. On the other hand, the adsorption efficiency (R%) of AC increased gradually at the beginning with increasing concentration of phenol. It decreased when the concentration increased further. This typical trend has been previously reported, and the reason can be mainly attributed to the saturated occupation of the adsorption sites of AC. In other words, at a lower concentration, the higher adsorption may be due to the presence of more available sites on the adsorbent than the number of phenol ions which are available in the solution. However, at higher concentrations, the number of phenol ions is relatively higher than available sites for adsorption [24]. The maximum adsorption percentage was determined at 30 mg/L as 69% for GAC_950_, 83% for PAC_800_, and 87% for PAC_1000_.

**Figure 7:**
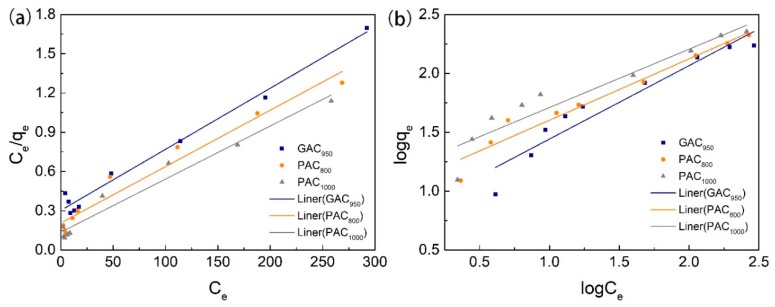
Fitting curves of adsorption on phenol through the Langmuir (**a**) and Freundlich (**b**) models.

The linear form of Langmuir and Freundlich isotherms of phenol on AC are shown in Figure 7 and the parameters of models are listed in Table 2. By comparing the coefficient of determination (*R^2^*), it can be concluded that the Langmuir model was more suitable to describe these sorption processes than the Freundlich model over the whole range of phenol concentrations; this indicated the homogeneous (monolayer) adsorption characteristics. In other words, AC had a uniform interfacial adsorption site for phenol [33]. PAC_1000_ had the highest adsorption capacity for phenol, and the maximum adsorption capacities calculated by the Langmuir isotherm were 246.31 mg/g. The next in line with a q_max_ of 232.02 mg/g was PAC_800_, and GAC_950_ was the lowest with an adsorption capacity of 214.13 mg/g. This can be explained by the fact that PAC_1000_ had the highest specific surface area and pore diffusion. Further, a possible explanation for the fact that the adsorption capacity of GAC_950_ was lower than PAC_800_ was the higher significance of pore diffusion and pore mouth closure in GAC [8,25].

### 3.4. The Effect of pH and Temperature on Phenol Adsorption

**Figure 8:**
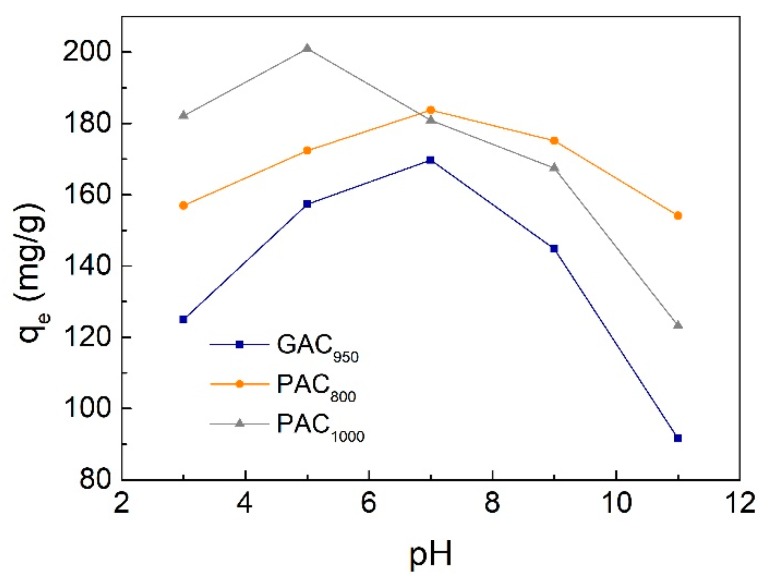
Effect of initial pH value on phenol adsorption onto AC.

The pH of the solution is a critical parameter affecting the adsorption process because it affects the surface charge of the adsorbents as well as the degree of ionization and speciation of pollutants. It is a common observation that the surface adsorbs anions favorably at lower pH due to the presence of H^+^ ions, whereas the surface is active for the adsorption of cations at higher pH due to the deposition of OH^−^ ions [34]. In this work, the effect of pH on the adsorption of phenol was investigated under five individual pH values of 3.0, 5.0, 7.0, 9.0, and 11.0. The initial concentration of phenol was 300 mg/L, the contact time was ≥420 min, and the adsorption temperature was controlled at 25 °C accordingly. As shown in Figure 8, the maximum adsorption was determined at pH 7 as 169.72 mg/g for GAC_950_ and 183.72 mg/g for PAC_800_. The maximum for PAC_1000_ was 200.96 mg/g at pH 5. The decrease in phenol adsorption as the pH dropped from 7(5) to 3 was mainly due to electrostatic repulsion. In detail, under acidic conditions, the pH_PZC_ (point of zero charge) of AC (GAC_950_ 7.4, PAC_800_ 7.6, and PAC_1000_ 5.7) were higher than the pH of solution, and there were many positive charges on the surface of AC. Further, the phenolic compounds were in the non-ionized forms, and the surface groups were either neutral or positively charged. The adsorption of water and phenol was competitive and the adsorbability of phenol was low. At pH >7(5), the decrease of phenol adsorption may have resulted from two reasons. First, the negative charges on the surface of AC increased with pH and phenol changed from molecular state to ionic state, which made the repulsion force between phenol ions and AC significant. Second, phenolate anions were more soluble in the aqueous solution, and stronger adsorbate–water bonds must be broken before adsorption can take place [7,20].

**Figure 9:**
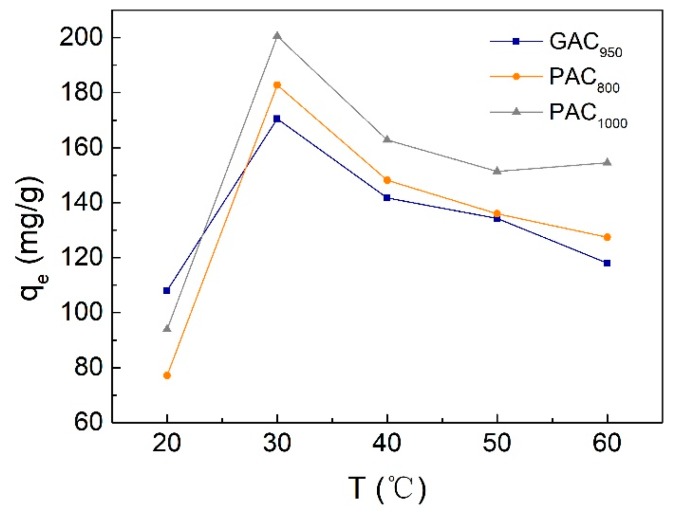
Effect of temperature value on phenol adsorption onto AC.

Temperature is another important parameter for removing phenol from wastewater, and it is desirable to obtain a high q_e_ under room temperature without additional energy consumption. Figure 9 represents the experimental results of temperature effect on phenol adsorption within the range of 20–60 °C. The adsorption capacity of AC for phenol increased with the increase of temperature when the temperature was lower than 30 °C; however, when the temperature was higher than 30 °C, the adsorption capacity of AC decreased with the increase of temperature. At 30 °C, the q_e_ values of GAC_950_, PAC_800_, and PAC_1000_, were 170.48, 182.77, and 200.49 mg/g, respectively. Because sorption is an exothermic process, it would be expected that an increase in temperature of the adsorbate–adsorbent system would result in decreased sorption capacity. However, if the adsorption process is controlled by the diffusion process (intraparticle transport-pore diffusion), the sorption capacity will show an increase with an increase in temperatures [31]. As has been shown earlier, the diffusion of adsorbate into pores of the sorbent was also a rate-controlling step. Therefore, the increase in sorption capacity with an increase in low temperature may be attributed to the diffusion process playing an important role. On the other hand, the active binding sites of AC were damaged and the binding forces between the phenol molecules and AC were weakened at a higher temperature; thus, the trend of desorption of phenol from the interface to the solution was increased [14,34].

### 3.5. The Effect of Humic Acid (HA) on Phenol Adsorption

**Figure 10:**
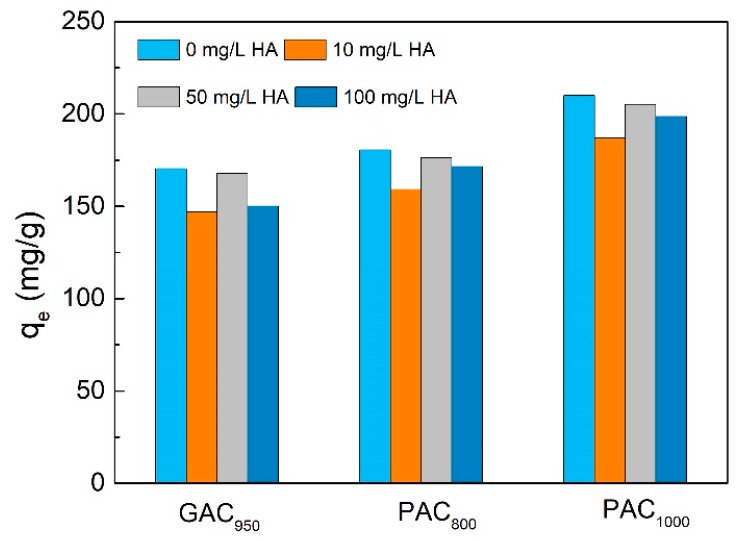
Effect of humic acid (HA) concentration on phenol adsorption onto AC.

The humic acid was combined with phenol to investigate the adsorption between humic acid (HA) and phenol for ACs in our work. As is shown in Figure 10, when the concentration of HA was 10 mg/L, q_e_ decreased from 170.39 to 146.96 mg/g, 180.58 to 159.15 mg/g, and 210.01 to 186.87 mg/g for GAC_950_, PAC_800_, and PAC_1000_, respectively, with decrease rates of 13.75%, 11.87%, and 11.02%, respectively. Then, as the concentrations of HA increased, the q_e_ value increased slowly; however, it was still lower than the q_e_ without HA. The adsorption capacity of phenol on AC was decreased in the presence of HA because of competition by HA with phenol, either site competition or pore/interstice blockage [35]. In this case, the humic acid had an average molecular weight of 1716 ± 478 (by number) or 3303 ± 904 g/mol (by mass), whereas phenol had a molecular weight of 94 g/mol. It was unlikely that the humic acid would be capable of occupying the small pores energetically preferred by the phenol molecules. It was speculated that hydrophilic groups such as carboxyl group, hydroxyl group, and phenolic hydroxyl group in HA molecular structure were easy to unite with the oxygen-containing groups of AC, forming complex on the AC surface and thus blocking the adsorption site of AC [36]. Moreover, HA behaved as polyelectrolytes in solution and changed the surface charge of AC by making AC more negative, which increase adsorption of phenol reasonably. Some researchers have also concluded that the increased adsorption is due to desorption of poreblocking phenol [37].

### 3.6. Desorption Study

**Figure 11:**
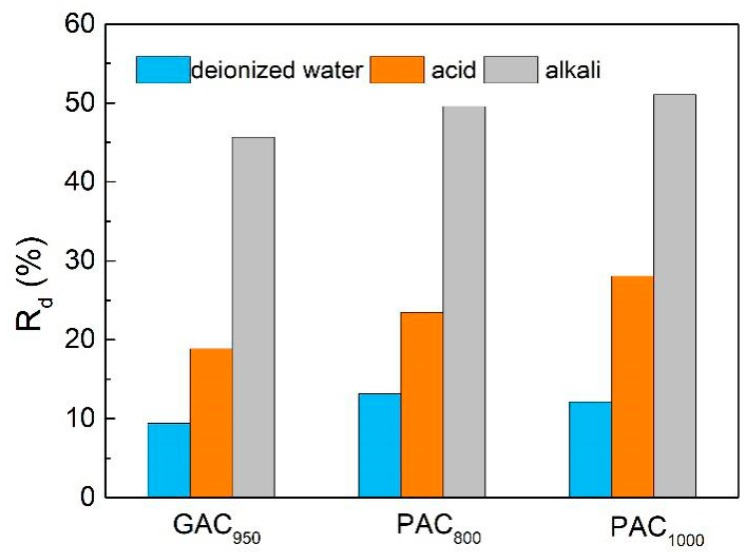
The phenol release percentage of AC.

Desorption studies help elucidate the mechanism of adsorption and recover the precious phenols, water, and adsorbent. Attempts were made to desorb phenol from the spent carbons using various solutions (deionized water/acid/alkali), and the results are shown in Figure 11. The release rate of AC in alkali was higher than that of acid and deionized water. This may be attributed to the formation of salt of phenol, which may have facilitated desorption of phenol from the carbon surfaces and weakened the interaction between phenol and AC [19]. Further, the desorption rates of phenol from PAC_800_ and PAC_1000_ in deionized water were 13% and 12%, respectively, whereas for GAC_950_ it was only 9%, which may have been caused by the small pore size of GAC_950_. Phenol has the high affinity of the compounds to AC, and it’s hard to desorption after it penetrates into the smaller pores of AC [38].

## 4. Conclusions

The results concluded that the feedstock and physical appearance had a great effect on the properties of commercial AC and that they would impact the capacity and mechanism of phenol adsorption on commercial AC. Although the surface area values of GAC_950_ were higher than PAC_8__00_, both adsorption rate and the adsorption capacity of GAC_950_ were found to be lower than PAC_8__00_. A possible explanation for this observation was the higher pore diffusion in PAC_8__00_. Compared with PAC_800_, PAC_1000_ was derived from coconut shell, possessing higher surface area and pore diffusion, and had the highest adsorption capacity for phenol. Additionally, the effects of several parameters, including the pH, temperature, initial phenol concentration, and contact time, were systematically investigated, and the desirable adsorption conditions, adsorption kinetics model, and equilibrium model for phenol were obtained. More importantly, the three commercial AC are suitable to be used for emergency treatment of phenol accidents because of their low desorption ratio in possible release conditions.

## Figures and Tables

**Table 1:** Kinetic parameters for phenol adsorption onto AC.

Model	AC		
	GAC_950_	PAC_800_	PAC_1000_
pseudo-first-order			
k_1_ (min^−1^)	0.0115	0.0062	0.0287
qe (mg/g)	133.62	51.09	52.04
*R^2^*	0.9348	0.9259	0.8535
pseudo-second-order			
k_2_ (g/mg/min)	2.01 × 10^−4^	7.94 × 10^−4^	1.59 × 10^−3^
q_e_ (mg/g)	175.44	175.44	212.77
*R^2^*	0.9861	0.9989	0.9998
Weber–Morris			
k_3_ (mg/g/min^1/2^)	6.3023	3.2536	2.4276
C	51.865	118.45	172.74
*R^2^*	0.9576	0.8809	0.7433

**Table 2:** Equilibrium parameters for phenol adsorption onto AC.

Model	AC		
	GAC_950_	PAC_800_	PAC_1000_
Langmuir			
k_L_ (L/mg)	0.0154	0.0209	0.0298
q_m_ (mg/g)	214.13	232.02	246.31
*R^2^*	0.9814	0.9545	0.9630
Freundlich			
k_F_ (mg/g)	6.581	12.051	16.385
n	1.603	1.919	2.018
*R^2^*	0.9141	0.9482	0.8915
